# Understanding the Strategies to Overcome Phosphorus–Deficiency and Aluminum–Toxicity by Ryegrass Endophytic and Rhizosphere Phosphobacteria

**DOI:** 10.3389/fmicb.2018.01155

**Published:** 2018-06-01

**Authors:** Patricio J. Barra, Sharon Viscardi, Milko A. Jorquera, Paola A. Duran, Alexander J. Valentine, María de la Luz Mora

**Affiliations:** ^1^Center of Plant, Soil Interaction and Natural Resources Biotechnology, Scientific and Technological Bioresource Nucleus, Universidad de La Frontera, Temuco, Chile; ^2^Departamento de Procesos Diagnósticos y Evaluación, Facultad de Ciencias de la Salud, Universidad Católica de Temuco, Temuco, Chile; ^3^Department of Botany and Zoology, Faculty of Science, Stellenbosch University, Stellenbosch, South Africa

**Keywords:** phosphobacteria, organic acids, phosphatases, malate dehydrogenase, acid soils, aluminium–toxicity, phosphorus–deficiency

## Abstract

Phosphobacteria, secreting organic acids and phosphatases, usually favor plant performance in acidic soils by increasing phosphorus (P) availability and aluminum (Al) complexing. However, it is not well-known how P-deficiency and Al-toxicity affect the phosphobacteria physiology. Since P and Al problems often co-occur in acidic soils, we have therefore proposed the evaluation of the single and combined effects of P-deficiency and Al-toxicity on growth, organic acids secretion, malate dehydrogenase (*mdh*) gene expression, and phosphatase activity of five Al-tolerant phosphobacteria previously isolated from ryegrass. These phosphobacteria were identified as *Klebsiella* sp. RC3, *Stenotrophomona* sp. RC5, *Klebsiella* sp. RCJ4, *Serratia* sp. RCJ6, and *Enterobacter* sp. RJAL6. The strains were cultivated in mineral media modified to obtain (i) high P in absence of Al–toxicity, (ii) high P in presence of Al–toxicity, (iii) low P in absence of Al–toxicity, and (iv) low P in presence of Al–toxicity. High and low P were obtained by adding KH_2_PO_4_ at final concentration of 1.4 and 0.05 mM, respectively. To avoid Al precipitation, AlCl_3_ × 6H_2_O was previously complexed to citric acid (sole carbon source) in concentrations of 10 mM. The secreted organic acids were identified and quantified by HPLC, relative *mdh* gene expression was determined by qRT-PCR and phosphatase activity was colorimetrically determined using p-nitrophenyl phosphate as substrate. Our results revealed that although a higher secretion of all organic acids was achieved under P–deficiency, the patterns of organic acids secretion were variable and dependent on treatment and strain. The organic acid secretion is exacerbated when Al was added into media, particularly in the form of malic and citric acid. The *mdh* gene expression was significantly up–regulated by the strains RC3, RC5, and RCJ6 under P–deficiency and Al–toxicity. In general, Al–tolerant phosphobacteria under P deficiency increased both acid and alkaline phosphatase activity with respect to the control, which was deepened when Al was present. The knowledge of this bacterial behavior *in vitro* is important to understand and predict the behavior of phosphobacteria *in vivo*. This knowledge is essential to generate smart and efficient biofertilizers, based in Al–tolerant phosphobacteria which could be expansively used in acidic soils.

## Introduction

Acidic volcanic soils are frequently characterized by high contents of total phosphorus (P), but sparingly bioavailable as orthophosphate anions (${\text{HPO}}_{4}^{2-}$ and H_2_${\text{PO}}_{4}^{-}$), which are required as nutrient for plants. Orthophosphate anions are strongly adsorbed on the surfaces of clay fractions and other particles, and colloids of acidic soils or precipitated as inorganic salts principally with aluminum (Al) and iron (Fe) (Redel et al., 2016). Under acidic soil conditions, the harmless Al compounds (such as aluminosilicates and oxides of Al) are solubilized into Al^3+^, which is generally toxic to microorganisms (Lemire et al., 2010) and plants (Wulff-Zottele et al., 2014). In vegetative tissues, low Al^3+^ concentrations are sufficient to affect cellular integrity at the root apex and thereby limiting water and nutrient uptake by sensitive plants (Kochian, 2003). Accordingly, it is desirable to develop environmentally friendly strategies, focused on increasing the efficiency by which plants can access the unavailable soil P forms in soils, as well as to develop tolerance strategies to alleviate the Al–toxicity to plants grown in acidic soils.

Higher plants have naturally developed strategies to survive in acidic soils, where the production and exudation of phosphatases and low-molecular-weight organic acids, such as citric, malic, oxalic and succinic, play a central role (Inostroza-Blancheteau et al., 2012; Chen and Liao, 2016). Root exudates stimulate growth and chemotaxis of microorganisms in the rhizosphere, resulting in a nutrient-rich microbial hot-spot where usually inhabit numerous beneficial bacteria, known as plant growth-promoting rhizobacteria (PGPR) (Richardson et al., 2009). Diverse studies have suggested the use of PGPR to improve P nutrition by plants, also named as phosphobacteria, because they can increase the orthophosphate availability to plants by secreting P–hydrolyzing enzymes (Jorquera et al., 2008; Patel et al., 2010) and organic acids (Vyas and Gulati, 2009; Sharon et al., 2016). While phosphobacteria are able of synthetizing and secreting both acid (ACP, EC 3.1.3.2) and alkaline (ALK, EC 3.1.3.1) phosphatases (Azcón-Aguilar and Barea, 2015), plants only can produce ACP (Spohn et al., 2015). In the rhizosphere, both ACP and ALK mineralize the P attached to organic compounds, whereas that organic acids are fundamental in solubilizing the P strongly adsorbed to soil complexes via ligand exchange (Richardson et al., 2009). The organic acids also solubilize P precipitated as inorganic Al– and Fe– salts via metal chelation in the rhizosphere, thus avoiding further toxic Al^3+^ uptake by plant (Richardson et al., 2009). Organic acids have therefore an important function in acidic soils, both in the P–solubilization and Al tolerance.

Organic acid production is catalyzed by various interrelated enzymes, such as malate dehydrogenase (MDH, EC 1.1.1.37.), which is encoded by the *mdh* gene. The MDH is an ubiquitous enzyme that catalyzes the reversible reduction of oxaloacetate to malate (Lü et al., 2012a). Some studies have demonstrated that both P–deficiency (Wang et al., 2014) and Al–toxicity (Chen et al., 2015) up–regulates *mdh* gene expression in plants, which has been related with an enhanced exudation of the organic acids, malate, and citrate (Ligaba et al., 2004). Therefore, the upregulation of *mdh* gene expression, may underpin an enhanced P–efficiency (Lü et al., 2012b) and Al–tolerance (Tesfaye et al., 2001) in acidic soils. Although plant *mdh* gene expression is known under Al and P stress, the *mdh* gene expression in PGPR under these adverse conditions has not been previously examined. Likewise, although organic acid secretion and phosphatase activity are characteristics which are widely described for soil bacteria, there is very little information available about their patterns of production and activity *in vitro*, under controlled conditions of P–deficiency and Al–toxicity. To date, the underlying mechanisms of bacterial adaptation to the environmental stressing factors, especially Al–toxicity, typically present in acidic soils remain poorly understood.

Many studies have suggested and demonstrated the agronomic potential of using phosphobacteria as a suitable and sustainable biotechnological alternative to increase P availability in acidic soils (Jorquera et al., 2008). However, it is still necessary to mechanistically understand the functional responses of phosphobacteria to these adverse soil conditions, and the genetic regulation of some of these key responses. Therefore, we hypothesized that Al–tolerant phosphobacteria use similar key metabolic strategies as plants, to overcome P–deficiency and Al–toxicity in soils. To confirm this hypothesis we have evaluated the single and combined effects of P–deficiency and Al–toxicity on growth, production of organic acids, *mdh* gene expression and phosphatase activity of five phosphobacteria previously selected by their ability to both solubilize and mineralize insoluble P forms, and tolerate high Al concentration (Mora et al., 2017).

## Materials and methods

### Bacterial strains

Five phosphobacteria strains identified as *Klebsiella* sp.RC3, *Stenotrophomonas* sp.RC5, *Klebsiella* sp. RCJ4, *Serratia* sp. RCJ6, and *Enterobacter* sp.RJAL6 were selected from a previous study by Mora et al. (2017). The strains were isolated from rhizosphere and endosphere of ryegrass (*Lolium perenne*) grown in an acidic volcanic soils (Andisol) from La Araucanía Region, in southern Chile (38° 50′ S, 72° 41′ W). All strains were selected based on Al tolerance (10 mM) and plant growth–promoting traits as described by Mora et al. (2017). The five phosphobacteria strains were deposited in the microbial collection CCCT (Colección Chilena de Cultivos Tipo) of Scientific and Technological Bioresource Nucleus BIOREN–UFRO, Temuco, Chile.

### DNA extraction and PCR reaction

Bacterial DNA of each strain was extracted from overnight culture in LB broth using a Gentra Puregene Yeast/Bact. Kit (Qiagen, Inc.) according to the manufacturer instructions. The *mdh* gene fragments were amplified by PCR using the primers set mdh2 (5′−GCG CGT AAG CCG GGT ATG GA−3′) and mdh4 (5′−CGC GGC AGC CTG GCC CAT AG−3′) as described Yap et al. (2004). Amplicons were sequenced in both sense by Macrogen, Inc. (Seoul, Korea). The consensus nucleotide sequences were compared with the GenBank database from the National Center for Biotechnology Information (NCBI) using BLAST tools (http://www.ncbi.nlm.nih.gov/BLAST). The nucleotide sequences of *mdh* gene segments were deposited in GenBank database under accession numbers MG023310–MG023319.

### Culture conditions

In order to test the effects of P–deficiency and Al–toxicity on selected phosphobacteria, a standard mineral culture medium (MCM) was prepared and modified from those described by Guida et al. (1991) and Appanna and St Pierre (1994), as follow. The MCM contained (L^−1^): 1.0 g NH_4_Cl, 1.0 g KCI, 0.01 g CaCl_2_ × 2H_2_O, 0.87 g K_2_SO_4_, 0.2 g MgSO_4_ × 7H_2_O, 54.4 mg KH_2_PO_4_ (only P source), 1.0 mg FeSO_4_ × 7H_2_O and trace elements (L^−1^): 10 μg H_3_BO_3_, 11.19 μg MnSO_4_ × H_2_O, 124.6 μg ZnSO_4_ × 7H_2_O, 78.22 μg CuSO_4_ × 5H_2_O, 10 μg MoO_3_ and. The solution of FeSO_4_ was sterilized by filtration (0.22 μm mesh), whereas the MgSO_4_ × 7H_2_O and trace elements solution were separately autoclaved and added aseptically to the salt sterile medium. The MCM was buffered with citrate buffer (pH 5.4) and the citrate from buffer was used as the only C source for bacterial growth at a final concentration of 4.0 g L^−1^. Four adapted MCM media were additionally formulated to obtain the following combinations: (i) high P in absence of Al–toxicity (P+ Al–), (ii) high P in presence of Al–toxicity (P+ Al+), (iii) low P in absence of Al–toxicity (P– Al–), and (iv) low P in presence of Al–toxicity (P– Al+). High and low P concentrations were defined according to Lidbury et al. (2016) by adding KH_2_PO_4_ at final concentration of 1.4 and 0.05 mM, respectively. To obtain high Al concentrations, the MCM was supplemented with AlCl_3_ × 6H_2_O in concentrations of 10 mM because this is the maximum tolerable Al concentration detected for these strains by Mora et al. (2017). To avoid Al precipitation, AlCl_3_ × 6H_2_O was complexed to the 4.0 g of citric acid prior to sterilization as described by Appanna and St Pierre (1994). No Al was added in both Al– media. The pH of all media was adjusted to 5.4 by diluting NaOH. The MCM with P+ Al– was considered as controls under optimal culture conditions.

The phosphobacteria strains were grown in 10 ml of standard MCM at 30°C on a rotary shaker at 150 rpm. Late exponential cells were harvested by centrifugation at 3,500 rpm for 5 min. The pellets were washed three times with sterile saline solution (0.85% NaCl), resuspended and diluted to a final optical density of 0.1 at 600 nm (OD_600_). Subsequently, aliquots of 50 μL from each bacterial suspension were inoculated (in triplicate) in 10 mL of each modified MCM media. All bacterial cultures were incubated at 30°C on a rotary shaker at 150 rpm. Growth was monitored as the change in optical density for 48 h at 600 nm using Multiskan™ GO Microplate Spectrophotometer (Thermo Fisher Scientific Inc.).

### Secretion of organic acids

All bacterial cultures were harvested during the late exponential phase of growth. For this, 2 mL aliquots were transferred to 1.5 mL tubes and centrifuged at 13,000 rpm for 1 min. Supernatants were filtered through 0.22 μm syringe filters and stored at −20°C until analysis. Then, 20 μL of each filtrate (in triplicate) was analyzed via high performance liquid chromatography (HPLC) (Hitachi Primaide, Japan), equipped with a UV– 210 nm detector. The organic acid separation was carried out on RP−18 150833 columns as described by Mora et al. (2017). The organic acids were identified by comparing their retention times and the peak areas of their chromatograms with those of standards for citric, malic, oxalic and succinic acid, by using Primaide System Manager Software. The organic acids determined here were selected because are the main organic acid exuded by *Lollium perenne* in acid soils (Rosas et al., 2011).

### Relative expression of malate dehydrogenase (*mdh*) gene

Relative expression of *mdh* gene was determined by quantitative PCR (qRT-PCR) as follow. Total RNA was isolated from 2 mL of each bacterial culture using TRIzol® reagent (Life Technologies™) and treated with RNase-free DNase I (New England, Biolabs) to eliminate DNA contaminations according to the manufacturer's instructions. The RNA concentrations and purity were measured spectrophotometrically using Multiskan™ GO (Thermo Fisher Scientific, Inc.). The RNA concentration was adjusted to 100 ng μL^−1^ and cDNA was synthesized by using High–Capacity cDNA Reverse Transcription Kit (Applied Biosystems, Thermo Fisher Scientific, Inc.) according to the manufacturer's instructions. Relative quantification was performed in an Applied Biosystems Step One™ Real–Time PCR System in 20 μL reaction mixtures containing PowerUp™ SYBR® Green master mix (Applied Biosystems, Life Technologies.), 1 μL of 1:10 dilution of the synthesized cDNA and 600 nM of each primer. PCR were performed in quadruplicate under the following conditions: an initial denaturing step at 95°C for 10 min and 40 cycles at 95°C for 30 s, 52°C for 30 s, and 60°C for 1 min. In this experiment, 16S rRNA was used as an endogenous control by using universal primer set Bac1369F (5′−CGG TGA ATA CGT TCY CGG−3′) and Prok1492R (5′−GGW TAC CTT GTT ACG ACT T−3′) (Suzuki et al., 2000). Data of target gene quantification was normalized using the endogenous control. The normalized values were subjected to a 2^−ΔΔCt^ method (Pfaffl, 2001) to estimate the fold change between endogenous control and target gene. The obtained data were transformed to log2 fold change.

### Phosphatase activity

Extracellular and intracellular cell–associated phosphatase activity of phosphobacteria were assayed at acid (pH 5.5) and alkaline (pH 8.0) pH by using p–nitrophenylphosphate (*p*–NPP) as substrate. Extracellular phosphatase activity was determined in the supernatants of bacterial culture media. Whereas, cell–associated phosphatase activity was determined in sonicated pellet as described by Patel et al. (2010). To phosphatase activity, 100 μL aliquots of supernatant or sonicated cell suspension were incubated with 5 μL of 0.115 M *p*–NPP, along with 5 μL of 0.1 M MgCl_2_, and 5 μL of 0.1 M Na–acetate buffer pH 5.5 for ACP activity, or with 0.1 M Tris–HCl buffer pH 8.0 for ALP activity. The reaction was incubated in dark at 30°C for 30 min, after which the reaction was stopped by adding 115 μL of 2 N NaOH. The amount p–nitrophenol (*p–*NP) released was determined by measuring absorbance at 405 nm in 96 wells microplates in a Multimodal Detector Synergy™ HT, BioTek. One unit of phosphatase was defined as the amount that releases 1 μmol of *p–*NP min^−1^. The phosphatase activity was expressed on a protein basis as mU mg^−1^ protein. Protein concentration was colorimetrically quantified by using the Bradford's method.

### Data analysis

The data were analyzed by ANOVA and the means were compared using the Tukey's multiple comparison test for mean separation. Correlations amongst organic acid production and *mdh* gene expression were tested with Spearman's nonparametric correlation analysis. In all analyses, differences at *P* ≤ 0.05 were considered as significant differences between treatments. The analyses were conducted using the IBM SPSS 21 software. In addition, a principal component analysis (PCA) plot was generated using the statistical software package R (http://cran.at.r-project.org).

## Results

### Bacterial growth

**Figure 1:**
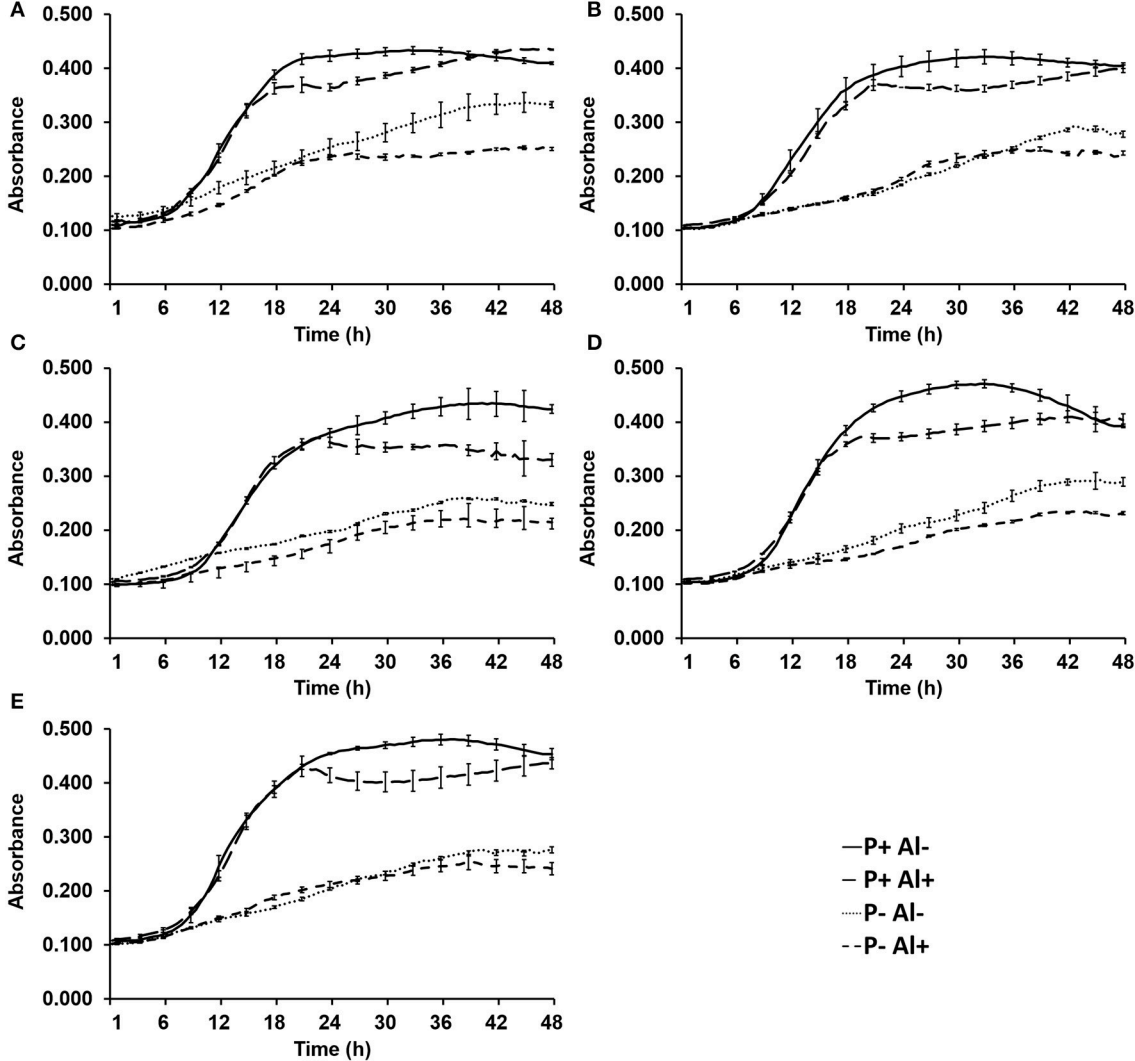
Growth of selected phosphobacteria strains **(A)** *Klebsiella* sp. RC3, **(B)** *Stenotrophomonas* sp. RC5, **(C)** *Klebsiella* sp. RCJ4, **(D)** *Serratia* sp. RCJ6, and **(E)** *Enterobacter* sp. RJAL6 in a modified mineral culture medium (MCM) with contrasting P and Al concentrations. P+: 1.4 mM KH_2_PO_4_; P–: 0.05 mM KH_2_PO_4_; Al+: 10 mM AlCl_3_ × 6H_2_O; Al–: without Al added.

The effect of P–deficiency and Al–toxicity on growth of five selected phosphobacteria are illustrated in Figure 1. The results revealed that the five phosphobacteria strains were able to grow in the modified MCM, even under P–deficiency (0.05 mM KH_2_PO) and Al addition (10 mM AlCl_3_ × 6H_2_O). In all tested strains, the exponential growth phase was not significantly (*P* ≤ 0.05) affected by Al when phosphobacteria were grown with high P concentration (1.4 mM KH_2_PO). However, the stationary growth phase showed lower cell density (OD_600_) and started before when the phosphobacteria were grown in presence of Al (18–24 h) than in control conditions (21–33 h). The Figure 1 also shows that the growth of all phosphobacteria strains was significantly (*P* ≤ 0.05) affected by P–deficiency. Thus, under P–deficiency the exponential growth phase was slower and the stationary growth phase was reached later (36–42 h), and a significantly (*P* ≤ 0.05) lower absorbance (0.210~0.320) compared with cultures with higher P concentration (0.360~0.480) was observed at OD_600_. Interestingly, adverse effect of P–deficiency on the growth of all phosphobacteria was more pronounced in presence of Al, particularly when they were grown in P– Al+ MCM. In Figure 1 and in Table S1 (available in the online Supplementary Material) it is also possible to observe that there are different degrees of tolerance to P–deficiency and Al–toxicity among the bacterial strains tested. Thus, after 24 h of incubation the strain *Klebsiella* sp. RCJ4 was the phosphobacteria less inhibited by Al–toxicity (P+Al+). However, this strain was gradually decreasing its relative growth with respect to control (P− Al+), to become the most inhibited strain at 36 and 48 h of growth (Table S1). In contrast, *Klebsiella* sp. RC3 and *Stenotrophomonas* sp.RC5 were the less inhibited strains by Al–toxicity at 36 and 48 h of growth, although they were among the most inhibited phosphobacteria at 24 h. Interestingly, four strains grown under Al–toxicity equaled the biomass of the controls after 48 h of incubation. Similarly, *Klebsiella* sp. RC3 and *Stenotrophomonas* sp.RC5 were the phosphobacteria that showed the highest tolerance to P–deficiency, both in presence and in absence of Al, since were the strains with lowest growth inhibition at 24, 36, and 48 h of incubation in P–, Al–, and in P– Al+ MCM. Meanwhile, *Klebsiella* sp. RCJ4, *Enterobacter* sp.RJAL6 and *Serratia* sp. RCJ6 were the strains that on average had a greater growth inhibition under P–deficiency, Al–toxicity and the combination of both factors, respectively. These findings have demonstrated that the growth of phosphobacteria is rather limited by P–deficiency than by Al–toxicity, at least in Al-tolerant phosphobacteria, which are well adapted to the presence of this toxic cation.

### Secretion of organic acids

In general terms, HPLC analysis (Figure S1; available in the online Supplementary Material) revealed that the selected phosphobacteria strains secreted all tested organic acids (Table 1), in at least one of the treatments. Under considered optimal culture conditions (P+ Al–), the patterns of organic acids secretion were very variable and depending on treatment and strain. However, when modified MCM was supplemented with Al (P+ Al+), coincidently all tested strains showed the secretion of succinic acid, at the same time as the other organic acids tested were almost not secreted. Similar patterns were observed when selected strains were gown under P–deficiency (P– Al–), where all tested strains showed the secretion of succinic acid. In addition, when this is compared to culture media with high (P+) and low P (P–) addition, a higher secretion of all organic acids was achieved under P−deficiency. Coincidently, the organic acid secretion is exacerbated when Al was added into media, particularly in malic and citric acids. Table 1 also shows that *Enterobacter* sp.RJAL6 was the strains that secreted the highest concentration of succinic acid and the only one that secreted citric acid under Al–toxicity (P−Al+). Whereas, under P−deficiency (P− Al–) the highest concentration the citric and malic acid, and succinic and oxalic acid were secreted by *Serratia* sp. RCJ6 and *Stenotrophomonas* sp. RC5, respectively. Coincidently, *Stenotrophomonas* sp. RC5 was also the strain that produced more citric acid when both stressing factors were present. Meanwhile, RJAL6 produced significantly higher concentration of malic acid in the P+ Al+ MCM. Thus, apparently the stimulation of the production and secretion of organic acids under the typical conditions present in acid soils is a transversal characteristic in plant–associated phosphobacteria, independent of the species. With respect to Spearman's correlation coefficients presented in Table S2 (available in the online Supplementary Material), malic acid secretion had a significant (*P* ≤ 0.05) positive correlation with oxalic and citric acid secretion under both Al–toxicity (P+Al+ and P–Al+) treatments and under P–deficiency (P– Al–), respectively. Meanwhile, the secretion of citric acid was negatively correlated with oxalic acid under both P−deficiency treatments (P−Al– and P−Al+) and with malic acid secretion only in the treatment with P−Al+. Likewise, the secretion of succinic acid was negatively correlated with malic and citric acid under P−deficiency (P– Al–) and positively correlated with the secretion of citric and oxalic acid under P+Al+ and P–Al+, respectively.

**Table 1:** Organic acid secretion by five selected phosphobacteria strains grown in mineral culture media (MCM) with contrasting P and Al concentrations.

**Strain^a^**	**Treatment^b^**	**Organic acid (mg L**^**−1**^**) ^C^**			
		**Oxalic acid**	**Malic acid**	**Citric acid**	**Succinic acid**
RC3	P+Al–	N.D.	26.65 ± 0.11 c	0.37 ± 0.01 e	N.D.
	P+Al+	N.D.	N.D.	N.D.	45.03 ± 1.48 c
	P–Al–	0.10 ± 0.01 d	0.06 ± 0.02 e	N.D.	24.15 ± 2.11 h
	P–Al+	0.02 ± 0.00 de	N.D.	29.42 ± 0.16 d	N.D.
RC5	P+Al–	1.30 ± 0.01 a	N.D.	N.D.	69.26 ± 0.10 a
	P+Al+	0.14 ± 0.01 d	1.12 ± 0.01 e	N.D.	40.39 ± 0.50 de
	P–Al–	1.30 ± 0.14 a	N.D.	N.D.	31.19 ± 0.50 g
	P–Al+	0.10 ± 0.01 d	N.D.	301.34 ± 11.46 b	42.82 ± 1.89 cd
RCJ4	P+Al–	N.D.	N.D.	N.D.	N.D.
	P+Al+	N.D.	N.D.	N.D.	36.59 ± 1.73 e
	P–Al–	N.D.	N.D.	3.02 ± 0.01 e	21.43 ± 0.66 h
	P–Al+	0.02 ± 0.00 e	20.00 ± 0.01 d	50.18 ± 4.72 c	N.D.
RCJ6	P+Al–	N.D.	N.D.	453.68 ± 0.38 a	N.D.
	P+Al+	N.D.	N.D.	N.D.	35.45 ± 1.10 f
	P–Al–	0.07 ± 0.01 d	0.43 ± 0.01 e	17.50 ± 2.84 d	3.92 ± 0.03 j
	P–Al+	0.51 ± 0.03 b	47.33 ± 0.27 b	0.56 ± 0.01 e	39.41 ± 1.06 def
RJAL6	P+Al–	N.D.	N.D.	N.D.	N.D.
	P+Al+	N.D.	N.D.	0.30 ± 0.01 e	59.81 ± 2.58 b
	P–Al–	0.06 ± 0.01 d	0.43 ± 0.01 e	2.66 ± 0.61 e	7.96 ± 1.09 i
	P–Al+	0.35 ± 0.01 c	63.00 ± 4.24 a	0.45 ± 0.11 e	43.23 ± 0.53 cd

a *Klebsiella sp. RC3, Stenotrophomonas sp. RC5, Klebsiella sp. RCJ4, Serratia sp. RCJ6, and Enterobacter sp. RJAL6*.

b *P+: 1.4 mM KH_2_PO_4_; P–: 0.05 mM KH_2_PO_4_; Al+: 10 mM AlCl_3_ × 6H_2_O; Al–: without Al added*.

C *Average ± standard error from three replicates. Different letters denote significant differences (P ≤ 0.05; Tukey's test) in the secretion of each organic acid among treatments and strains. N.D, not detectable*.

### Relative expression of *mdh* gene

**Figure 2:**
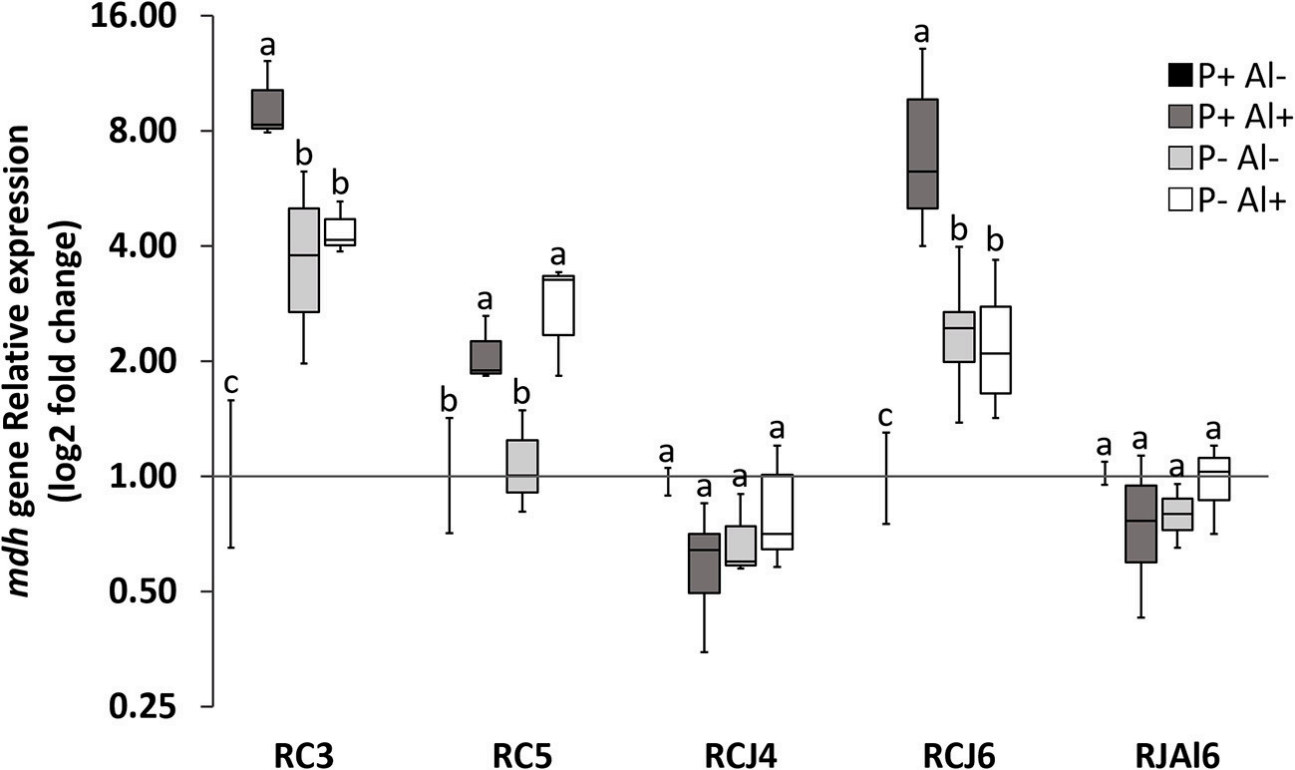
Relative quantification of malate dehydrogenase (*mdh*) gene of five phosphobacteria: *Klebsiella* sp. RC3, *Stenotrophomonas* sp. RC5, *Klebsiella* sp. RCJ4, *Serratia* sp. RCJ6, and *Enterobacter* sp. RJAL6 grown in a modified mineral culture medium (MCM) with contrasting P and Al concentrations. P+: 1.4 mM KH_2_PO_4_; P–: 0.05 mM KH_2_PO_4_; Al+: 10 mM AlCl_3_ × 6H_2_O; Al–: without Al added. Different letters on bars denote significant differences (*P* ≤ 0.05; Tukey's test) in phosphatase activity among treatments and strains.

**Figure 3:**
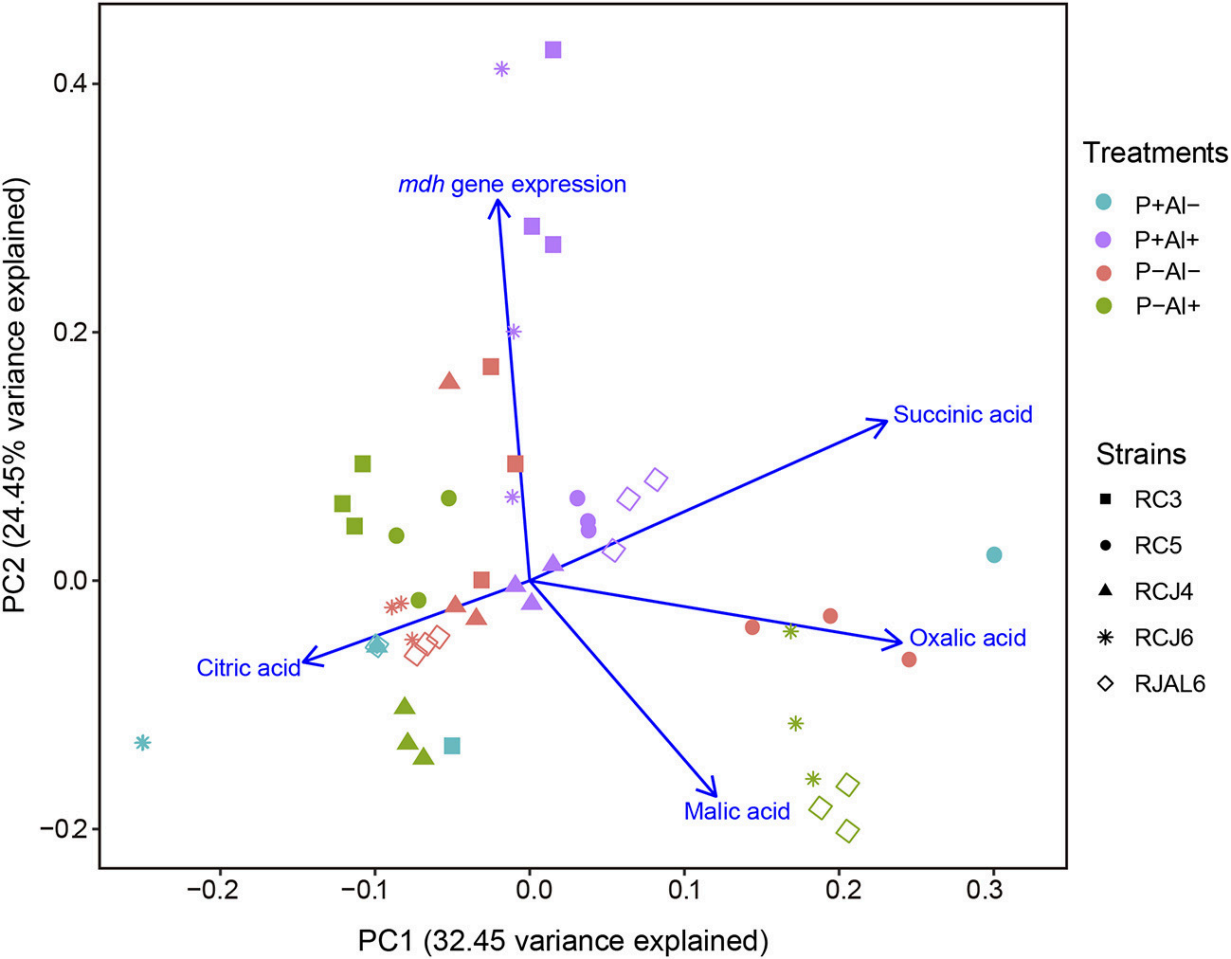
Principal component analysis (PCA) of organic acid secreted and malate dehydrogenase (mdh) gene expression of five phosphobacteria: *Klebsiella* sp. RC3, *Stenotrophomonas* sp. RC5, *Klebsiella* sp. RCJ4, *Serratia* sp. RCJ6, and *Enterobacter* sp. RJAL6 grown in a modified mineral culture medium (MCM) with contrasting P and Al concentrations. P+: 1.4 mM KH_2_PO_4_; P–: 0.05 mM KH_2_PO_4_; Al+: 10 mM AlCl_3_ × 6H_2_O; Al–: without Al added.

Reverse transcription followed by qPCR was used to determine the relative expression of *mdh* gene by selected phosphobacteria strains subjected to P–deficiency and Al–toxicity. The results illustrated in Figure 2, revealed that *mdh* gene expression was significantly (*P* ≤ 0.05) up–regulated under P–deficiency, Al–toxicity and the combination of both factors with respect to treatment control (P+ Al–) by the strains *Klebsiella* sp. RC3 and *Serratia* sp. RCJ6. Meanwhile, Al–toxicity both in the presence and absence of P (P+ Al+ and P– Al+), caused significant (*P* ≤ 0.05) up–regulation of *mdh* gene expression in *Stenotrophomonas* sp. RC5. In contrast, P–deficiency and/or Al–toxicity did not significantly (*P* ≤ 0.05) affected *mdh* gene expression of the strains *Klebsiella* sp. RCJ4 and *Enterobacter* sp. RJAL6. Our results also revealed that *mdh* gene expression was negatively correlated with malate secretion under P– Al+ treatment (Table S2; available in the online Supplementary Material). With respect to the PCA (Figure 3) its first two dimensions explained 57.0% of the total variation, with principal component 1 (PC1) accounting for 32.5% and principal component 2 (PC2) for 24.5% of the variance. Interestingly, the results illustrated in the PCA shows that in general the groups were joined according to treatment rather than species.

### Phosphatase activity

**Figure 4:**
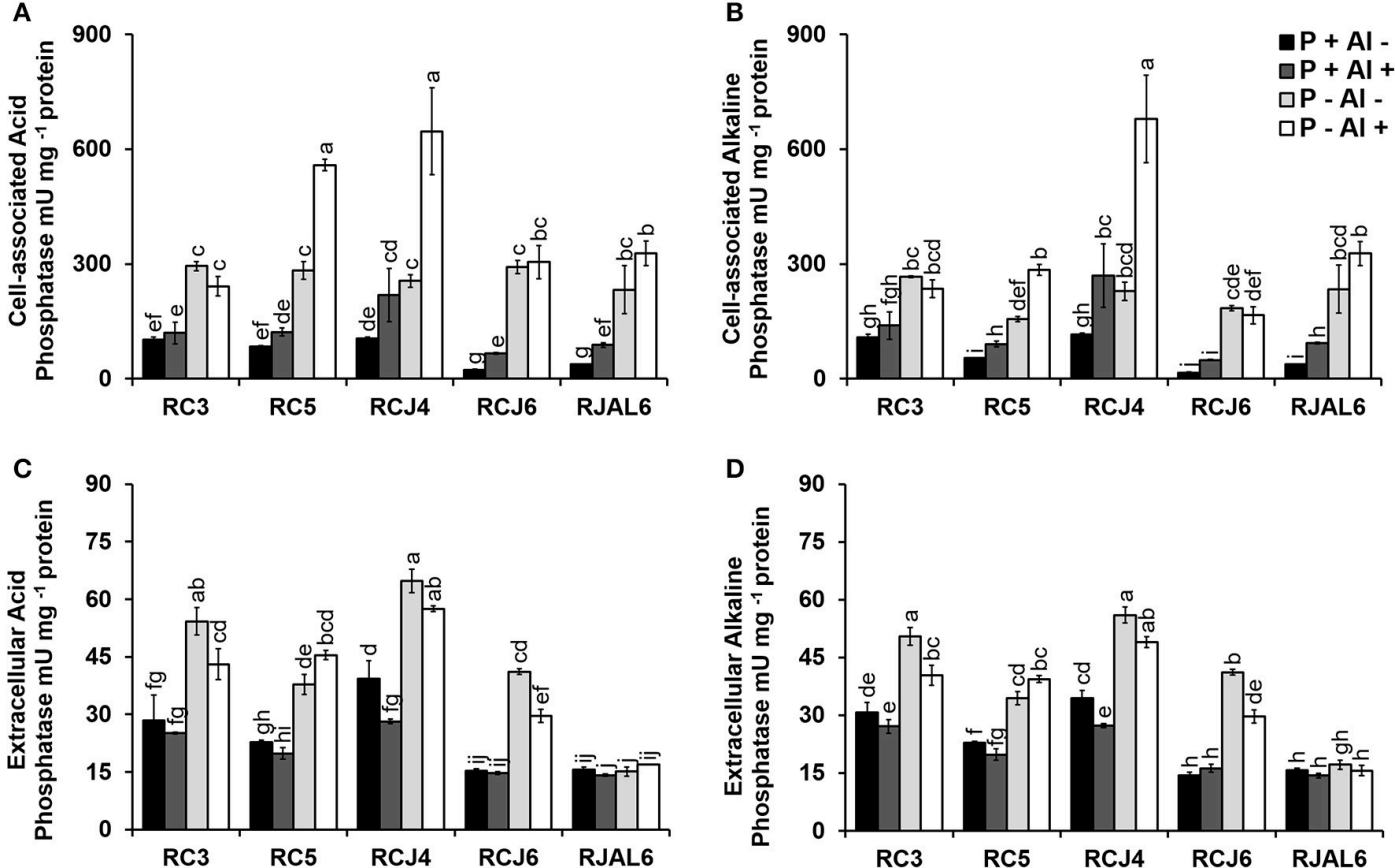
Acid and alkaline phosphatase activity by five phosphobacteria *Klebsiella* sp. RC3, *Stenotrophomonas* sp. RC5, *Klebsiella* sp. RCJ4, *Serratia* sp. RCJ6, and *Enterobacter* sp. RJAL6 grown in a modified mineral culture medium (MCM) with contrasting P and Al concentrations. P+: 1.4 mM KH_2_PO_4_; P–: 0.05 mM KH_2_PO_4_; Al+: 10 mM AlCl_3_ × 6H_2_O; Al–: without Al added. **(A)** Cell–associated acid phosphatase, **(B)** cell–associated alkaline phosphatase, **(C)** extracellular acid phosphatase, and **(D)** extracellular alkaline phosphatase. Different letters on bars denote significant differences (*P* ≤ 0.05; Tukey's test) in phosphatase activity among treatments and strains.

The Figure 4 illustrates bacterial both cell–associated and extracellular acid (ACPase) and alkaline (ALPase) phosphatase activities. The results showed considerable variation in phosphatase activities among treatments and strains. In general, phosphobacteria grown under P–deficiency increased significantly (*P* ≤ 0.05) cell–associated and extracellular ACPase with respect to control treatment (Figures 4A,C). Similar observations were obtained in ALPase activity, where significantly (*P* ≤ 0.05) higher cell–associated and extracellular ALPase activities were attributed to P–deficiency treatments (Figures 4B,D). Whereas, under Al toxicity (P+ Al+) almost all strains increased significantly (*P* ≤ 0.05) the cell-associated acid and alkaline phosphatase activity, which, however, did not result in a significant (*P* ≤ 0.05) improvement in extracellular phosphatase activity. The higher cell–associated ACPase activity under P–deficiency and Al toxicity was registered by *Klebsiella* sp. RCJ4 followed by *Stenotrophomonas* sp.RC5 (Figure 4A), while higher extracellular ACPase activity was also registered by *Klebsiella* sp. RCJ4 followed by *Klebsiella* sp. RC3 under P–deficiency P−deficiency (Figure 4C). The higher cell–associated ALPase activity was registered by *Klebsiella* sp. RCJ4 under P−deficiency and Al toxicity (Figure 4B), while the extracellular ALPase activity higher was registered by *Klebsiella* sp. RCJ4 followed by *Klebsiella* sp. RC3 under P–deficiency RC3 under P−deficiency (Figure 4D). In contrast, the lower phosphatase activities were mostly registered by *Enterobacter* sp. RJAL6, particularly for extracellular phosphatase independently of treatments (Figures 4C,D). These results demonstrate that phosphobacteria efficiently respond to the P shortage by increasing its production and secretion of both alkaline and acid phosphatase.

## Discussion

Deficiency of P and Al–toxicity often coexist in acidic soils affecting negatively to diverse organisms, including bacteria (Appanna and St Pierre, 1994). The interactions of Al–P are very relevant in physicochemical properties of Chilean acidic volcanic soils (Mora et al., 2017). However, the effect of Al–P interactions on growth and performance of soil bacteria have been scarcely studied, due mainly to the fact that Al spontaneously precipitates with P at acid pH. In the present study we described a mineral culture medium (MCM), which proved to be suitable to evaluate the response of phosphobacteria to P deficiency, Al–toxicity and the combination of both environmental stressors *in vitro*. The response to these conditions was tested in five phosphobacteria strains, which were selected particularly based on their ability to efficiently tolerate high Al concentrations (Mora et al., 2017) and to grow in P shortage (this study), but also for possessing several plant growth promoting (PGP) traits, such as production of indole acetic acid and siderophore, as well as, 1-aminocyclopropane-1-carboxylate deaminase, phytate mineralizing and P solubilization activities (Mora et al., 2017). In this context, by minimum inhibitory concentration (MIC) assay, it was revealed that after 4 days of bacterial incubation in a culture media supplemented with 0–40 mM Al(NO_3_)_3_ the selected phosphobacteria strains were able to tolerate Al concentration up to 10 mM (Mora et al., 2017). Similarly, we determined by a MIC assay (data no shown) that bacterial growth was inhibited by 50% at 0.05 mM KH_2_PO_4_ (P concentration that was chosen to supplement the P- MCM) and almost completely inhibited at 0.003 mM KH_2_PO_4_. The Al–tolerance and the ability to growth with low P availability, along with the PGP traits, makes the selected bacteria strains good candidates to be used in sustainable agriculture in acidic soils.

The obtained data in the present study have demonstrated that high Al concentrations reduced the growth rate after 24 h (4.6–18.8%), and induced a growth stationary phase in Al–tolerant phosphobacteria strains, as already described for rhizobia and *Pseudomonas fluorescens* strains (Keyser and Munns, 1979; Wood, 1995). The negative effect of Al on bacterial growth, although not fully elucidated, is due to the fact that this toxic cation mediates a malfunction of the cell membranes and cell walls (Illmer and Erlebach, 2003), binds to DNA, thereby affecting DNA synthesis (Piña and Cervantes, 1996) and interferes with central metabolism by competition with Fe and Mg (Lemire et al., 2010) resulting in an energy deficit, disturbed aerobic metabolism and increased oxidative stress (Hamel and Appanna, 2001; Mailloux et al., 2008). Despite this potential negative effect, the biomass of selected strains after 48 h of incubation under Al–toxicity tends to be equal to that of the controls, which corroborates that the select bacteria are naturally adapted to the presence of this metal.

In line with expectations, our results have also revealed that P deficiency significantly decreased the bacterial growth, even more severely than Al–toxicity. It has been extensively described that P deficiency produce a significant decrease in bacterial growth rates (Munns and Keyser, 1981; Wood and Cooper, 1984). These results are clearly explained because P is an essential macro-nutrient for all organisms, by virtue of its requirements for the synthesis of many biomolecules, such as ATP, phospholipids and nucleic acids. It is noteworthy that the detrimental effect of P–deficiency on bacterial growth was exacerbated by the presence of Al in the culture media. Despite to the inherent difficulties to study the joint effect of P and Al on bacterial growth, a previous study published by Appanna and St Pierre, (1994) described that the Al–tolerant bacteria *P. fluorescens* decreased by 10–40% its cellular yield in the presence of 3–15 mM Al when was grown in P deficient media. These authors concluded that the Al tolerance of *P. fluorescens* is dependent on the P concentration of the medium, because when P is in excess (Appanna et al., 1994) the Al is immobilized as an exocellular gelatinous insoluble residue, subsequently identified as phosphatidylethanolamine (Appanna and St Pierre, 1996), a phospholipid typically present in biological membranes. In contrast, under P–deficiency the Al was localized in soluble metabolite(s) in the supernatant (Appanna and St Pierre, 1994), which apparently derive from citric acid, an Al–chelating organic acid (Hamel et al., 1999). Therefore, phosphate and organic acids appears to be important ligand involved in Al detoxification (Appanna and St Pierre, 1994).

Organic acids secreted into the rhizosphere have traditionally been implicated in important soil processes, including the release and uptake of nutrients by microorganisms and plants (Jones, 1998; Azcón-Aguilar and Barea, 2015; Sharon et al., 2016), and the detoxification of metals by plants (Chen and Liao, 2016). The release of organic compounds by soil bacteria is a well–documented phenomenon (Vyas and Gulati, 2009; Gulati et al., 2010) and many aspects of their metabolic machinery have already been investigated (Singh et al., 2009). Nevertheless, the factors influencing their production and secretion have yet to be fully elucidated. This is because organic acids synthesis is a complex and intricate process involving the activities of multiple enzymes and the expression of their corresponding coding genes (Yin et al., 2015), which can be downregulated or up–regulated depending on the requirements of the bacterial cell (Hamel and Appanna, 2001). Indeed, it is well known that situations such as metal toxicity and the consequent oxidative stress induce several metabolic reconfigurations in order to ensure energy production and bacterial survival (Mailloux et al., 2008). Our study has confirmed that the phosphobacteria tested here, have the ability to metabolize the Al–citrate complex to obtain energy and produce biomass. It should also be noted that all phosphobacteria strains were able to secrete the main organic acids that are also exuded by *L. perenne*, their host plant (Rosas et al., 2011; Mora et al., 2017). However, the concentration and types secreted organics acids are highly variable and dependent on bacterial strain and the culture conditions to which they were subjected (Al–toxicity, P–deficiency or both). Even phosphobacteria strains belonging to the same genera (*Klebsiella* sp. RC3 and *Klebsiella* sp. RCJ4) and subjected to identical conditions, exhibited different patterns of organic acid secretion as described by Vyas and Gulati (2009). Although the tested phosphobacteria do not follow a common pattern of secretion, larger concentrations and more varied composition of organic acids were secreted with the combination of P–deficiency and Al–toxicity compared with less stressed culture conditions. Thus, the secretion of oxalic, succinic and malic acids by *Serratia* sp. RCJ6 and *Enterobacter* sp. RJAL6 and citric and malic acid by *Klebsiella* sp. RCJ4 and citric acid by *Klebsiella* sp.RC3 and *Stenotrophomonas* sp.RC5 is augmented when are subjected to P–deficiency and Al–toxicity. Previous studies in *P. fluorescens* have corroborated that Al–citrate is translocated into the cell and metabolized intracellularly to be used as substrate in the production of other important Al–chelating organic compounds, such as oxalate (Appanna et al., 2003a; Singh et al., 2009) and citrate (Appanna et al., 2003b). Curiously, although citrate or Al–citrate were the only C source, the secretion of this organic acid was generally enhanced under stress conditions. This finding could be due to a reconfiguration of bacterial metabolic pathway that leads to the production of polycarboxylic aluminophore citrate derivative, which is involved in the sequestration of Al as proposed in the models described by Appanna et al. (2003b) and Lemire et al. (2008). Similarly, Mora et al. (2017) reported that the same phosphobacteria tested here, formed Al–chelating siderophores as evidenced by fluorescence emission assessed by confocal microscopy. It should be noted that the strains showing the highest tolerance to P–deficiency and Al–toxicity (that is, *Klebsiella* sp. RC3 and *Stenotrophomonas* sp. RC5) coincidentally were the strains with the highest production of citric acid under this condition. These results could be explained because the stability constants for Al–organic acid complexes are higher for citrate and oxalate than for malate and succinate (Hue et al., 1986; Poschenrieder et al., 2015).

Previous works has already demonstrated the secretion of oxalic, citric, malic and succinic acid by several phosphobacteria strains (*Arthrobacter, Bacillus, Serratia, Chryseobacterium, Pseudomonas*, and *Delftia*) in response to P–deficiency *in vitro* (Chen et al., 2006; Vyas and Gulati, 2009; Gulati et al., 2010). These phosphobacteria strains showed the ability to solubilize considerable amounts of tricalcium phosphate (Chen et al., 2006; Vyas and Gulati, 2009; Gulati et al., 2010), and to favor the growth, and uptake and accumulation of macro-nutrients (N, P, and K) in maize (Vyas and Gulati, 2009; Gulati et al., 2010). In similar way, oxalic, malic, succinic, and/or citric acids have also been implicated in Al–detoxification by various cellular systems (Hamel and Appanna, 2003; Hamel et al., 2004). In bacteria, augmented oxalic acid production, via increasing the activity of the enzyme isocitrate lyase (Hamel et al., 2004), have been described as the main strategy to detoxify high Al concentrations by the soil Al tolerant bacteria, *P. fluorescens*. In addition, the activities of citrate synthase, malate synthase and malate dehydrogenase are also increased by *P. fluorescens* in response to Al–toxicity (Lemire et al., 2010). According to our knowledge, this is the first report comparing the patterns of organic acid secretion by bacteria under the combined effects of P–deficiency and Al–toxicity.In relation to *mdh* gene expression, our results showed that *mdh* gene expression is dependent on the strain and do not follow a common pattern of expression when phosphobacteria are subjected to P–deficiency and Al–toxicity. However, three phosphobacteria strains (*Klebsiella* sp. RC3, *Stenotrophomonas* sp. RC05, and *Serratia* sp. RCJ6) increased the *mdh* gene expression under P–deficiency and Al–toxicity, which was negatively correlated with malate secretion. Previous reports have associated an enhanced organic acids exudation with increased expression of *mdh* gene both in bacteria (Lü et al., 2012a) and plants (Tesfaye et al., 2001; Lü et al., 2012b). Thus, *Escherichia coli* overexpressing a mitochondrial malate dehydrogenase (mMDH) gene from *Penicillium oxalicum* C2 increased the secretion of malate, citrate, oxalate, lactate, and acetate in culture media, which in turn improved the tricalcium phosphate solubilizing ability of the bacteria (Lü et al., 2012a). Similarly, increased synthesis and exudation of malate have been found in tobacco (Wang et al., 2010) overexpressing *mdh* gene from *E. coli*. In contrast to these previous reports, our results show no evident association between an increased *mdh* gene expression and organic acid exudation by phosphobacteria. Further molecular studies are yet necessaries to fully understand the complex network of organic acids regulation, production and secretion by phosphobacteria in acid soils. In this regard, it is possible that MDH enzyme activity is not only regulated by the gene expression of *mdh*, but also by post-translational modification of the protein.

Many researchers have described the important role of bacterial phosphatases (both ACP and ALP) in P cycling in terrestrial ecosystems for plants (Fraser et al., 2015, 2017; Acuña et al., 2016). In order to provide an alternative P source to biological systems, phosphatase hydrolyzes phosphomonoesters with a wide substrate specificity (Fraser et al., 2015). All phosphobacteria strains tested have previously been described as P–mineralizing bacteria by Mora et al. (2017), which is in agreement with our findings that demonstrated both cell–associated and extracellular phosphatase activity. In general, our study showed an increase in ACP and ALP activities under P–deficiency. Our observations are also consistent with recent findings that have described under P limiting conditions increased bacterial phosphatase activity, both ACP and ALP (Fraser et al., 2015; Spohn et al., 2015), which is reduced as a result of inorganic P fertilization (Spohn et al., 2015). Molecular studies have confirmed that labile P in soil is negatively correlated with bacterial non–specific acid (*pho*C) and alkaline (*pho*D) phosphatase gene abundance and phosphatase activity (Fraser et al., 2017). In addition, an early study described that fast–growing rhizobial strains contained high levels of ALP activity under P–limited conditions (Smart et al., 1984). Our study has also provided evidence that the ACP and ALP activities by two strains, *Stenotrophomonas* sp.RC5, *Klebsiella* sp. RCJ4, are even more pronounced when Al is present in the culture media. Although there are now many studies available about the effects of Al on bacterial phosphatase activity, Kunito et al. (2016) revealed significant influences of the exchangeable Al concentration, as well as pH, on the microbial ACP activity in acidic forest soils. Sonicated cell lysates of phosphobacteria showed higher activities than the extracellular compartment. However, cell–associated phosphatases may be more related to other cell metabolic functions than playing any role in extracellular P mineralization (Menezes-Blackburn et al., 2013). Therefore, our study suggest that phosphobacteria have developed similar strategies to deal with P–deficiency and Al–toxicity that their host plant.

## Conclusion

Here, we describe the responses of Al–tolerant phosphobacteria to P–deficiency and Al–toxicity, principal stressors present in acidic volcanic soils. Although the growth of phosphobacteria is negatively affected for the combination of both stressors, they tolerate and survive these adverse conditions. This is achieved by the synthesis and secretion of organic acids and the activities of both acid and alkaline phosphatase enzymes. The secretion of organic acids is generally enhanced under stressful conditions, although the patterns of secretion and concentrations, are very variable and dependent on treatment and strain. Furthermore, the bacterial phosphatase activity is more strongly increased by P–deficiency, than by Al–toxicity. In order to generate novel and efficient phosphobacteria–based biofertilizers, for extensive application in acidic soils, a continued investigation of bacterial responses *in vitro*, is essential to understand the regulation of these organisms in soils.

## Author contributions

PB, MJ, and MM designed the research and supervised the study. PB, MJ, PD, and AV contributed intellectually to data analysis. PB and SV performed laboratory work. PB and MM wrote the manuscript. PB, MJ, and PD designed Tables and Figures. All authors revised the manuscript and approved the final version.

### Conflict of interest statement

The authors declare that the research was conducted in the absence of any commercial or financial relationships that could be construed as a potential conflict of interest. The reviewer OV-L and handling Editor declared their shared affiliation.

## Supplementary material

Figure S1 HPLC chromatograms of standard organic acids and of the five phosphobacteria grown in control mineral culture medium (MCM) supplemented with 1.4 mM KH_2_PO_4_ and without Al added (P+ Al–).

Table S1 Percentage (%) of growth inhibition with respect to the control (P+ Al–) of five phosphobacteria *Klebsiella* sp. RC3, *Stenotrophomonas* sp. RC5, *Klebsiella* sp. RCJ4, *Serratia* sp. RCJ6, and *Enterobacter* sp. RJAL6 grown in a modified mineral culture medium (MCM) with contrasting P and Al concentrations. P+: 1.4 mM KH_2_PO_4_; P–: 0.05 mM KH_2_PO_4_; Al+: 10 mM AlCl_3_ × 6H_2_O; Al–: without Al added.

Table S2 Spearman's rank correlation matrix for organic acid secretion and malate dehydrogenase (*mdh*) gene expression.
