# Supplementary figures and images for: Understanding the Strategies to Overcome Phosphorus–Deficiency and Aluminum–Toxicity by Ryegrass Endophytic and Rhizosphere Phosphobacteria
Source: Front Microbiol. 2018 Jun 1;9:1155. doi: 10.3389/fmicb.2018.01155 (PMC5992465; doi:10.3389/fmicb.2018.01155)

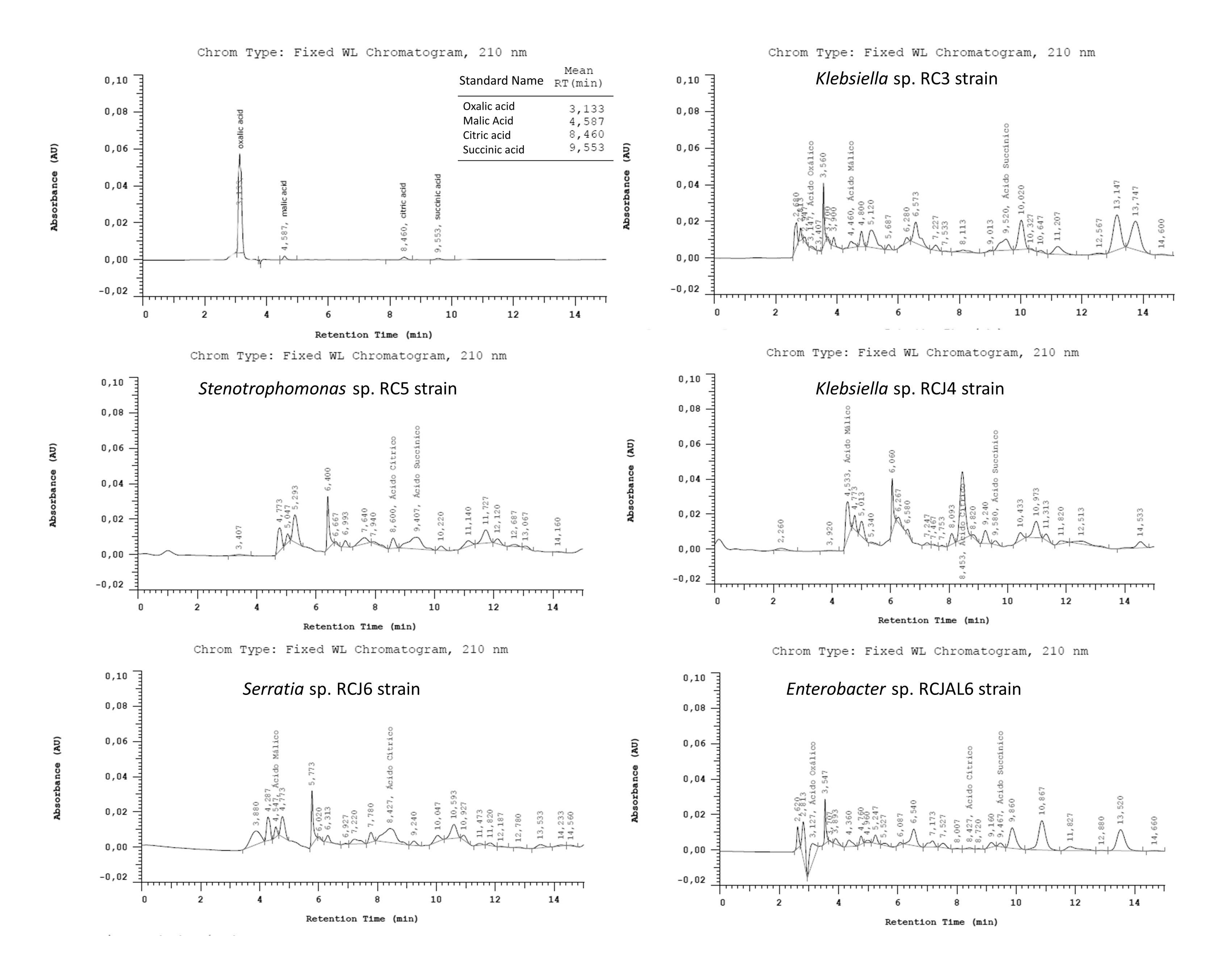

Supplement: Figure S1 — HPLC chromatograms of standard organic acids and of the five phosphobacteria grown in control mineral culture medium (MCM) supplemented with 1.4 mM KH2PO4 and without Al added (P+ Al–). [file Image_1.TIF]
