# Supplementary material for: Understanding the Strategies to Overcome Phosphorus–Deficiency and Aluminum–Toxicity by Ryegrass Endophytic and Rhizosphere Phosphobacteria
Source: Front Microbiol. 2018 Jun 1;9:1155. doi: 10.3389/fmicb.2018.01155 (PMC5992465; doi:10.3389/fmicb.2018.01155)
Supplement: Table S1 — Percentage (%) of growth inhibition with respect to the control (P+ Al–) of five phosphobacteria Klebsiella sp. RC3, Stenotrophomonas sp. RC5, Klebsiella sp. RCJ4, Serratia sp. RCJ6, and Enterobacter sp. RJAL6 grown in a modified mineral culture medium (MCM) with contrasting P and Al concentrations. P+: 1.4 mM KH2PO4; P–: 0.05 mM KH2PO4; Al+: 10 mM AlCl3 × 6H2O; Al–: without Al added. [file Table_1.DOCX]

|  | **RC3** | | |  | **RC5** | | |  | **RCJ4** | | |  | **RCJ6** | | |  | **RJAl6** | | |
| --- | --- | --- | --- | --- | --- | --- | --- | --- | --- | --- | --- | --- | --- | --- | --- | --- | --- | --- | --- |
| **Tiempo**  **(h)** | **P+Al+** | **P-Al-** | **P-Al+** |  | **P+Al+** | **P-Al-** | **P-Al+** |  | **P+Al+** | **P-Al-** | **P-Al+** |  | **P+Al+** | **P-Al-** | **P-Al+** |  | **P+Al+** | **P-Al-** | **P-Al+** |
| 3 | -2.6 | -10.7 | 7.0 |  | -6.3 | 1.9 | -0.9 |  | -6.7 | -18.3 | -0.3 |  | -7.3 | -0.3 | 2.9 |  | -5.5 | 4.0 | 3.1 |
| 6 | -3.9 | -9.2 | 6.3 |  | -4.8 | 2.5 | 0.3 |  | -8.2 | -25.3 | -1.3 |  | -9.1 | -3.2 | 3.2 |  | -6.4 | 3.1 | 5.0 |
| 9 | -2.2 | 7.3 | 22.8 |  | 3.4 | 16.7 | 17.8 |  | -8.8 | -21.3 | 0.8 |  | -10.4 | 8.5 | 12.7 |  | -4.5 | 15.0 | 15.2 |
| 12 | 5.5 | 27.8 | 40.7 |  | 11.3 | 38.6 | 40.1 |  | 0.6 | 9.7 | 26.3 |  | 0.4 | 37.3 | 39.6 |  | 7.3 | 40.3 | 38.9 |
| 15 | 0.8 | 38.8 | 46.5 |  | 8.3 | 50.2 | 50.7 |  | -0.5 | 34.0 | 45.5 |  | 1.4 | 52.2 | 55.1 |  | 1.4 | 53.0 | 50.3 |
| 18 | 6.4 | 44.3 | 47.9 |  | 8.3 | 56.5 | 55.1 |  | -1.5 | 45.3 | 53.8 |  | 6.6 | 57.1 | 62.0 |  | 2.0 | 56.2 | 51.5 |
| 21 | 11.4 | 43.7 | 45.7 |  | 4.8 | 56.9 | 55.1 |  | -0.4 | 47.0 | 55.4 |  | 13.1 | 57.5 | 63.4 |  | 4.3 | 56.9 | 53.0 |
| 24 | 13.8 | 40.0 | 44.3 |  | 9.5 | 54.4 | 51.7 |  | 4.6 | 48.2 | 54.2 |  | 16.8 | 54.7 | 62.2 |  | 8.9 | 55.1 | 53.3 |
| 27 | 11.8 | 38.0 | 44.5 |  | 11.6 | 50.6 | 46.0 |  | 10.1 | 46.2 | 51.3 |  | 17.8 | 53.5 | 59.0 |  | 13.1 | 52.6 | 52.6 |
| 30 | 10.1 | 34.8 | 45.2 |  | 13.4 | 47.7 | 44.2 |  | 13.3 | 43.6 | 49.7 |  | 17.6 | 51.6 | 57.1 |  | 14.5 | 50.4 | 51.5 |
| 33 | 8.4 | 31.0 | 45.1 |  | 14.0 | 43.3 | 42.8 |  | 15.8 | 43.6 | 49.3 |  | 16.6 | 48.8 | 55.6 |  | 15.1 | 47.7 | 50.0 |
| 36 | 5.5 | 27.3 | 44.2 |  | 11.8 | 39.9 | 41.1 |  | 16.7 | 41.4 | 48.9 |  | 13.9 | 43.2 | 53.3 |  | 14.6 | 45.7 | 48.8 |
| 39 | 1.4 | 22.9 | 42.7 |  | 9.4 | 34.6 | 39.7 |  | 19.8 | 40.5 | 49.3 |  | 9.7 | 37.2 | 49.0 |  | 13.2 | 43.3 | 47.3 |
| 42 | -2.5 | 20.6 | 40.5 |  | 6.1 | 30.4 | 41.1 |  | 20.3 | 40.6 | 50.2 |  | 4.7 | 32.8 | 46.0 |  | 10.3 | 42.5 | 47.8 |
| 45 | -4.9 | 18.7 | 39.0 |  | 3.4 | 29.4 | 40.0 |  | 22.6 | 40.9 | 50.0 |  | -0.5 | 28.1 | 42.7 |  | 6.5 | 41.5 | 46.7 |
| 48 | -6.1 | 18.8 | 38.8 |  | 1.4 | 31.4 | 40.0 |  | 22.2 | 41.5 | 49.5 |  | -2.8 | 26.5 | 41.2 |  | 3.8 | 39.1 | 46.8 |
