# Supplementary material for: Understanding the Strategies to Overcome Phosphorus–Deficiency and Aluminum–Toxicity by Ryegrass Endophytic and Rhizosphere Phosphobacteria
Source: Front Microbiol. 2018 Jun 1;9:1155. doi: 10.3389/fmicb.2018.01155 (PMC5992465; doi:10.3389/fmicb.2018.01155)
Supplement: Table S2 — Spearman's rank correlation matrix for organic acid secretion and malate dehydrogenase (mdh) gene expression. [file Table_2.DOCX]

| **Treatment** |  | **Oxalic acid** | **Malic acid** | **Citric acid** | **Succinic acid** | *mdh* **gene expression** |
| --- | --- | --- | --- | --- | --- | --- |
| **P+ Al-** | **Oxalic acid** | 1.000 | - | - | - | - |
|  | **Malic acid** | -0.248 | 1.000 | - | - | - |
|  | **Citric acid** | -0.392 | 0.400 | 1.000 | - | - |
|  | **Succinic acid** | 0.993** | -0.246 | -0.389 | 1.000 | - |
|  | *mdh* **gene expression** | - | - | - | - | - |
|  |  |  |  |  |  |  |
| **P+ Al+** | **Oxalic acid** | 1.000 | - | - | - | - |
|  | **Malic acid** | 0.976** | 1.000 | - | - | - |
|  | **Citric acid** | -0.247 | -0.246 | 1.000 | - | - |
|  | **Succinic acid** | -0.036 | -0.049 | 0.684* | 1.000 | - |
|  | *mdh* **gene expression** | -0.008 | 0.010 | -0.449 | -0.018 | 1.000 |
|  |  |  |  |  |  |  |
| **P- Al-** | **Oxalic acid** | 1.000 | - | - | - | - |
|  | **Malic acid** | -0.122 | 1.000 | - | - | - |
|  | **Citric acid** | -0.554* | 0.517* | 1.000 | - | - |
|  | **Succinic acid** | 0.493 | -0.790** | -0.819** | 1.000 | - |
|  | *mdh* **gene expression** | 0.475 | 0.144 | -0.109 | -0.079 | 1.000 |
|  |  |  |  |  |  |  |
| **P- Al+** | **Oxalic acid** | 1.000 | - | - | - | - |
|  | **Malic acid** | 0.640** | 1.000 | - | - | - |
|  | **Citric acid** | -0.538* | -0.782** | 1.000 | - | - |
|  | **Succinic acid** | 0.613* | 0.382 | -0.208 | 1.000 | - |
|  | *mdh* **gene expression** | -0.215 | -0.582* | 0.150 | -0.079 | 1.000 |

* Represents statistically significant correlation (P ≤ 0.05), **represent statistically significant correlation (P ≤ 0.01).
